# Worldwide insect declines: An important message, but interpret with caution

**DOI:** 10.1002/ece3.5153

**Published:** 2019-04-05

**Authors:** Benno I. Simmons, Andrew Balmford, Andrew J. Bladon, Alec P. Christie, Adriana De Palma, Lynn V. Dicks, Juan Gallego‐Zamorano, Alison Johnston, Philip A. Martin, Andy Purvis, Ricardo Rocha, Hannah S. Wauchope, Claire F. R. Wordley, Thomas A. Worthington, Tom Finch

**Affiliations:** ^1^ Department of Zoology, Conservation Science Group University of Cambridge Cambridge UK; ^2^ Department of Zoology, Insect Ecology Group University of Cambridge Cambridge UK; ^3^ Department of Life Sciences Natural History Museum London UK; ^4^ School of Biological Sciences University of East Anglia Norwich UK; ^5^ Department of Environmental Science, Institute for Wetland and Water Research Radboud University Nijmegen The Netherlands; ^6^ Cornell Lab of Ornithology Cornell University Ithaca New York; ^7^ RSPB Centre for Conservation Science The Royal Society for the Protection of Birds Sandy UK

**Keywords:** entomofauna, invertebrates, population trends, systematic review

## Abstract

A recent paper claiming evidence of global insect declines achieved huge media attention, including claims of “insectaggedon” and a “collapse of nature.” Here, we argue that while many insects are declining in many places around the world, the study has important limitations that should be highlighted. We emphasise the robust evidence of large and rapid insect declines present in the literature, while also highlighting the limitations of the original study.

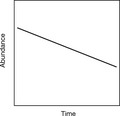

Ecologists and conservationists have long studied global declines in biodiversity, but insects—despite their abundance and diversity—are underrepresented in such assessments. Sánchez‐Bayo and Wyckhuys (2019) recently compiled and reviewed long‐term insect surveys from the peer‐reviewed literature. They report high average rates of decline in entomofauna and suggest 40% of the world's insect species could go extinct within decades.

We welcome this detailed focus on insect declines: their consistent underrepresentation is a shortcoming of the ecological literature, and it is essential that we improve and synthesize our knowledge, particularly given widespread anthropogenic threats. Many insect populations are undergoing rapid and worrying declines (Hallmann et al., 2017), which could have serious impacts on ecosystem function. However, we suggest the approach used in the review has four important limitations that could affect the conclusions: (a) biased search terms, (b) geographic biases, (c) incorrect estimation of extinction risks and rates, and (d) qualitative assignment of drivers to trends that was sometimes inaccurate, ignoring detail in the original work.

First, the authors’ aim was to compile “all long‐term insect surveys conducted over the past 40 years that are available through global peer‐reviewed literature databases,” but their inclusion of [declin*] as a required search term biases their evidence toward surveys that report population declines. Incorporating studies into the review which report increasing or stable populations could alter its conclusions about average trends. As an example, a study of Auchenorrhyncha declines is included in the review, while a similar piece of research published in the same year, by the same authors, in the same country finds increases in Heteroptera, but is not included (Schuch, Bock, Krause, Wesche, & Schaefer, 2012; Schuch, Wesche, & Schaefer, 2012). Similarly, the review of honey bees (*Apis mellifera*) mentions declines in the USA, Australia, and Europe, but not the global increase in the number of Western honey bee hives (IPBES, 2016).

Second, the acknowledged geographic bias toward North America and Europe means it is not appropriate to title the paper “Worldwide declines.” Given high spatial heterogeneity in threats and species’ distributions, it is difficult to extrapolate the results of better‐studied regions—which are unlikely to be representative—to other parts of the globe (Gonzalez et al., 2016). Bee responses to land‐use change, and global trends in other taxa such as birds, mammals, and amphibians, vary in magnitude and/or direction between North America and Europe and the rest of the world (Amano et al., 2018; Hoffmann et al., 2011; De Palma et al., 2016; Stuart et al., 2004). Therefore, it is unlikely that insect declines will be homogenous everywhere.

Third, when estimating the prevalence of species extinctions and extinction risk (Table 1 in the original paper), the authors misapply the IUCN Red List criteria by treating local, national, or regional population declines and extinctions as though they were global, and by omitting the criteria's stipulation that population declines need to have been rapid and recent (within the last decade or, if longer, three generations) for a species to qualify as threatened. Local declines of 80% over a century, for instance, should not be equated with global declines of 80% within the last ten years.

Finally, the authors attributed the trends to different threats using information from the original studies. Causal threats are challenging to identify, and some of the reported threats are simply postulated, rather than explicitly tested. For example, Conrad, Warren, Fox, Parsons, and Woiwod (2006) are cited as evidence that agricultural intensification, pesticide pollution, afforestation, and climate warming have all driven declines in British macro‐moths; however, that paper's discussion states clearly that “The causes of long‐term trends identified in this study are yet to be assessed in detail.” Although polling papers for their suggestions of causes of decline is valuable, it is not the same as synthesizing quantitative evidence. This matters because it could cause errors and bias in our understanding, leading to poorly informed management decisions.

As conservation scientists, we strive to communicate honest and accurate messages about our knowledge of the natural world. There is strong evidence that many insect populations are under serious threat from multiple pressures and are indeed declining in many places (Hallmann et al., 2017). We do not doubt the existence of such declines, but we must also be clear about the limitations of each study and dataset. While we believe the authors’ study is a useful review of insect population *declines* in North America and Europe, it should not be used as evidence of global insect population *trends* and threats. Future studies should employ robust, unbiased search terms; clear inclusion criteria; and explore publication, geographic, and taxonomic biases, perhaps within a formal meta‐analytic framework. Particularly given the high‐profile of this issue, results should be interpreted carefully and communicated with sensitivity to public perception. We hope our paper will stimulate further research, building on Sánchez‐Bayo and Wyckhuys's important work to more fully characterize changes in and threats to insect populations.

## CONFLICT OF INTEREST

Authors have no competing interests to declare

## AUTHOR CONTRIBUTIONS

BIS, TF, TAW, and PAM conceived the idea. BIS wrote the first draft of the manuscript. All authors contributed equally to subsequent edits.

## Data Availability

There are no data used in this article.
